# New Advances and Perspectives in Ophthalmology: Progress and Modern Challenges Toward Personalized Eye Care

**DOI:** 10.3390/jpm16060333

**Published:** 2026-06-22

**Authors:** Livio Vitiello

**Affiliations:** Eye Unit, “Luigi Curto” Hospital, Azienda Sanitaria Locale Salerno, 84035 Polla, Italy; livio.vitiello@gmail.com

## 1. Recent Developments in Ophthalmology

Over the past two decades, ophthalmology has been reshaped by the convergence of high-resolution multimodal imaging, pharmacological innovation and the broader paradigm of personalized medicine. Swept-source optical coherence tomography (OCT), OCT angiography, ultra-widefield retinal imaging and high-frequency ocular ultrasound now enable in vivo, micrometer-scale phenotyping of virtually every ocular structure [1], while artificial intelligence (AI) and deep learning turn these imaging outputs into quantitative biomarkers suitable for automated risk stratification [2,3]. In parallel, the therapeutic armamentarium has expanded from intravitreal anti-VEGF biologics—now available in longer-acting formulations and individualized treat-and-extend regimens [4]—to gene and cell therapies for inherited retinal dystrophies [5,6], and to increasingly refined cataract, refractive and oculoplastic platforms. Underlying these advances is a conceptual shift: ophthalmic diseases are no longer treated as homogeneous entities, but rather as heterogeneous clusters of phenotypes, each with their own risk profiles and optimal management.

## 2. Knowledge Gaps Addressed by This Special Issue

Despite this progress, several important gaps remain: real-world long-term data on individualized anti-VEGF regimens are still limited; ROP screening is inequitably distributed and logistically demanding; AI-based screening tools often lack prospective validation and safe-failure mechanisms [7]; IOL calculation remains problematic in post-refractive eyes [8]; predictors of surgical success in strabismus are poorly standardized; postural and dynamic components of intraocular pressure (IOP) are rarely integrated into routine glaucoma risk assessment; ocular imaging biomarkers of systemic disease are still being defined [9]; subclinical dysfunction in “inactive” orbitopathy is underdiagnosed; and the nutritional management of age-related macular degeneration (AMD) has evolved beyond the original AREDS framework without a consensus on individualized protocols. The sixteen contributions collected in this Special Issue directly address these gaps and sketch a coherent roadmap toward more personalized ophthalmic care.

## 3. Key Findings and Contributions

### 3.1. Retinal Vascular Disease and Anti-VEGF Personalization

Diabetic retinopathy (DR) remains a paradigmatic setting for personalized medicine. Zhou et al. (contribution 1) report 1-year outcomes of the VOYAGE extension study: in patients who had completed the PANORAMA trial and received 2 mg intravitreal aflibercept every 16 weeks as needed, less frequent dosing between PANORAMA exit and VOYAGE enrollment was associated with a measurable deterioration of the Diabetic Retinopathy Severity Scale score, best-corrected visual acuity and central subfield thickness, which subsequently stabilized or improved once regular, individualized treatment resumed. These findings are real-world evidence that anti-VEGF schedules must be tailored to ongoing disease activity. Ivanescu et al. (contribution 2) profiled 105 patients with type 1 or type 2 diabetes (77 with diabetic macular edema) in Western Romania, describing a high-risk cohort with median age 65 years, 15-year mean disease duration, HbA1c 7.5% and universal comorbidities (hypertension 100%, dyslipidemia 62.3%)—reinforcing the need for screening pathways integrated with systemic risk stratification.

### 3.2. Retinopathy of Prematurity: From Universal Screening to Tailored Pathways

Two complementary studies address retinopathy of prematurity. Christodoulou et al. (contribution 3) evaluated incidence, treatment patterns and risk factors for type 1 ROP in a national tertiary NICU in Cyprus, translating local epidemiology into a personalized screening-to-therapy pathway. Al-Abaiji et al. (contribution 4) demonstrated the feasibility of telescreening in Denmark using the ICON GO^®^ widefield camera operated by a non-physician healthcare professional: across 415 sessions in 165 neonates, on-site/off-site agreement was almost perfect for overall outcome (κ = 0.82) and substantial for stage and disease (κ = 0.69), with treatment needed in 3.6% of children. These findings provide evidence that delegated, tele-supported screening is safe, scalable, and a concrete step toward equitable neonatal care.

### 3.3. Artificial Intelligence and Glaucoma Screening

Hitzl et al. (contribution 5) leveraged a longitudinal dataset of 6889 subjects, with 585 completing an average 11.1-year follow-up period, to train two neural-network models equipped with a “reject option” that allows the algorithm to withhold a prediction in ambiguous cases. The clinical and economic rationale is to identify, already at baseline, the large proportion of screened individuals who remain free of open-angle glaucoma for up to 10 years and can therefore safely be excluded from intensive monitoring—a concrete translation of precision screening into everyday practice.

### 3.4. Precision in Refractive, Cataract and Strabismus Surgery

Tailored surgical planning is the focus of two studies. Lin et al. (contribution 6) compared the calibration accuracy of the Symfony extended depth-of-focus (EDOF) intraocular lens in eyes with and without previous LASIK, combining optical biometry with the SRK/T formula in non-LASIK eyes and Haigis-L in post-LASIK eyes—a clinically actionable framework for IOL power selection in complex refractive histories. Lino, de Aguiar and Cunha (contribution 7) retrospectively analyzed 258 children (mean age 11.2 years) with basic-type intermittent exotropia or divergence excess who underwent bilateral lateral rectus recession, identifying pre-operative predictors of post-operative alignment stability and refining the individualized surgical dosing needed to improve on the historical ∼75% success rate.

### 3.5. Intraocular Pressure, Biometrics and Glaucoma Biomechanics

De Bernardo et al. (contribution 8) measured IOP across sitting, supine, prone and standing postures in 124 eyes of 62 healthy subjects: the prone–supine differential was the largest (+2.24 mmHg; *p* < 0.001), followed by supine–sitting (+1.30 mmHg), while standing produced a significant IOP decrease relative to supine (−1.43 mmHg). Such postural profiles, which a single in-office measurement cannot capture, argue for a more individualized interpretation of IOP in selected glaucoma suspects. Gioia et al. (contribution 9) correlated the corrected axial length—a refinement addressing the systematic biometric error of cataractous eyes—with choroidal thickness, refining the quantitative framework for myopic and degenerative risk stratification.

### 3.6. Imaging Biomarkers and the Ocular–Systemic Interface

Three contributions use imaging biomarkers as windows onto systemic disease. Isleyen et al. (contribution 10) applied swept-source OCT to 67 patients with vasovagal syncope versus 61 controls, demonstrating a significantly thicker choroid in affected subjects without changes in peripapillary retinal nerve fiber layer (RNFL), showing that the choroidal vasculature is sensitive to autonomic dysregulation. Lixi et al. (contribution 11) reviewed the spectrum of ocular manifestations of patent foramen ovale, from transient visual disturbances and migraine with aura to oculomotor deficits and rare endogenous endophthalmitis from paradoxical embolism, reinforcing the need for a multidisciplinary ophthalmology–cardiology–neurology pathway. In 67 patients with inactive Graves’ orbitopathy, Lixi, Giannaccare and co-authors (contribution 12) combined orbital ultrasound with the Red Filter Test and detected latent diplopia in 49.25% of subjects despite no reported double vision—a strong case for sensitivity-based reassessment in a disease too often labeled “burnt out”.

### 3.7. Genomics and Precision Medicine in Inherited Retinal Disease

Two contributions address the genomic frontier of personalized ophthalmology. Dhivagaran et al. (contribution 13) provide a comprehensive review of precision medicine in inherited retinal diseases (IRDs), tracing the evolution from clinical phenotyping to next-generation sequencing (NGS)-based diagnostics and mutation-specific therapies. The review details how NGS has transformed the diagnostic landscape by enabling precise identification of causative variants across the more than 300 genes implicated in IRDs, and examines the patient-selection protocols—integrating genetic confirmation, structural retinal evaluation and genetic counseling—that underpin targeted interventions such as voretigene neparvovec (Luxturna) for RPE65-related disease. The authors also map the remaining barriers to broader adoption: high costs, uneven access to specialist services, and the interpretive complexity of variants of uncertain significance. Bakhamees et al. (contribution 14) complement this perspective with a cross-sectional survey of 115 ophthalmologists across Saudi Arabia, assessing knowledge, attitudes and practices toward genomic medicine. While 99% correctly recognized autosomal recessive inheritance and 90% endorsed the clinical value of genetic testing, confidence in practical tasks such as test selection and pre-test counselling was low (mean 4.7/10), and only 9% answered DNA base-pairing questions correctly. Taken together, these two contributions delineate a critical gap between the rapid maturation of genomic tools and the readiness of the clinical workforce to deploy them—and underscore the urgency of integrating genetics into ophthalmology training programs.

### 3.8. Diagnostic Imaging and Tailored Medical Therapy

Two narrative reviews complete this Special Issue. Coppola et al. (contribution 15) synthesized the literature on ocular ultrasound for optic neuropathies, emphasizing its bedside availability and particular value in patients unable to undergo magnetic resonance imaging, while acknowledging the methodological heterogeneity that still characterizes the field [10,11,12]. D’Angelo et al. (contribution 16) reviewed the evolving evidence on oral supplementation in AMD—the third leading cause of global visual impairment—mapping the interplay between oxidative stress, neuroinflammation and ocular ischemia, and discussing AREDS-based formulations alongside emerging compounds such as astaxanthin and melatonin. Their synthesis supports the move from one-size-fits-all supplementation toward individualized antioxidant strategies.

## 4. Future Research Directions

The contributions collected here open several avenues that will shape the next phase of personalized ophthalmology. First, imaging biomarkers—from choroidal thickness and RNFL to corrected axial length and extraocular muscle measurements—need cross-platform standardization, with consensus on acquisition protocols, reference ranges and reproducibility thresholds, before they can reliably guide individual decisions. Second, machine learning models should undergo prospective, multicenter validation in ethnically and demographically heterogeneous populations, ideally with confidence-aware outputs and human-in-the-loop oversight. Third, the integration of telemedicine, AI and portable imaging must be evaluated not only for diagnostic accuracy but also for cost-effectiveness and equity of access, particularly in vulnerable populations. Fourth, evidence on postural IOP, subclinical ocular motility dysfunction and ocular signs of systemic disease calls for the systematic integration of ophthalmology into cardio-, neuro- and endocrine-ophthalmology pathways. Fifth, pharmacological and nutritional management should move beyond population-average recommendations toward genotype-, phenotype- and biomarker-guided regimens for anti-VEGF therapy in DR/DME, IOL selection in complex refractive histories and antioxidant supplementation in AMD. Finally, patient-reported outcomes and adherence metrics must be prospectively incorporated into personalized protocols, so that success is defined not only by anatomical and functional endpoints but also by what matters most to patients.

## 5. Concluding Remarks

Collectively, the sixteen contributions in this Special Issue trace a coherent trajectory for contemporary ophthalmology: broader and earlier screening underpinned by telemedicine and AI; finer phenotyping supported by multimodal imaging and advanced biometrics; and more individualized therapeutic choices, from anti-VEGF regimens and IOL calculations to surgical dosing and antioxidant supplementation. The work presented here offers concrete building blocks for a genuinely personalized ophthalmic practice—and a clear agenda for the studies that will follow.

## References

[1] Wong Q.Y., Sim R., Ang M. (2026). Anterior Segment Optical Coherence Tomography with Angiography for the Cornea and Ocular Surface. J. Clin. Med..

[2] Parravano M., Cennamo G., Di Antonio L., Grassi M.O., Lupidi M., Rispoli M., Savastano M.C., Veritti D., Vujosevic S. (2024). Multimodal imaging in diabetic retinopathy and macular edema: An update about biomarkers. Surv. Ophthalmol..

[3] Rispoli M., Cennamo G., Antonio L.D., Lupidi M., Parravano M., Pellegrini M., Veritti D., Vujosevic S., Savastano M.C. (2023). Practical guidance for imaging biomarkers in exudative age-related macular degeneration. Surv. Ophthalmol..

[4] Menna F., De Luca L., Meduri A., Baldascino A., Lupo S., Vingolo E.M. (2026). New Clinical Trials and Therapeutic Advances with Faricimab and Aflibercept 8 mg in Neovascular Age-Related Macular Degeneration: A Durability-Oriented Comparative Perspective. Biomedicines.

[5] Ekemiri K., Daniel E., Seemongal-Dass R., Ekemiri C., Victor V., Amobi M., Adebo O., Tekalign T. (2026). Advanced therapeutic approaches for inherited retinal diseases: An umbrella review. BMJ Open.

[6] Britten-Jones A.C., Jin R., Gocuk S.A., Cichello E., O’Hare F., Hickey D.G., Edwards T.L., Ayton L.N. (2022). The safety and efficacy of gene therapy treatment for monogenic retinal and optic nerve diseases: A systematic review. Genet. Med..

[7] Savastano M.C., Rizzo C., Fossataro C., Bacherini D., Giansanti F., Savastano A., Arcuri G., Rizzo S., Faraldi F. (2025). Artificial intelligence in ophthalmology: Progress, challenges, and ethical implications. Prog. Retin. Eye Res..

[8] Cione F., De Bernardo M., Di Stasi M., De Luca M., Albano R., Rosa N. (2024). Lens Factor Choice in IOL Power Calculation after Laser Refractive Surgery: The Right Constant for Advanced Lens Measurement Approach (ALMA). J. Clin. Med..

[9] Crincoli E., Savastano M.C., Savastano A., Carlà M.M., Giannuzzi F., Catania F., Kilian R., Rizzo C., Rizzo S. (2026). Artificial intelligence and neurodegeneration: State of the art and explainability. Int. J. Retin. Vitr..

[10] De Bernardo M., Vitiello L., Rosa N. (2020). A-scan ultrasonography and optic nerve sheath diameter assessment during acute elevations in intra-abdominal pressure. Surgery.

[11] De Bernardo M., Vitiello L., Capone M., Rosa N. (2020). A-scan ultrasonography and optic nerve sheath diameter evaluation in children with acute liver failure. Liver Int..

[12] De Bernardo M., Vitiello L., Rosa N. (2019). Optic nerve sheath diameter ultrasonography in differentiation of ischemic and hemorrhagic strokes. Am. J. Emerg. Med..

